# Lack of Genetic Diversity in Newly Sequenced Porcine Circovirus Type 1 Strains Isolated 20 Years Apart

**DOI:** 10.1128/genomeA.00156-14

**Published:** 2014-04-10

**Authors:** Kata Tombácz, Robert Patterson, Sylvia S. Grierson, Dirk Werling

**Affiliations:** a Molecular Immunology Group, Department of Pathology and Pathogen Biology, Royal Veterinary College, Hatfield, United Kingdom; b Animal Health and Veterinary Laboratories Agency, Weybridge, United Kingdom

## Abstract

The complete genome sequences of a porcine circovirus type 1 (PCV1) strain isolated in 1990 and one isolated in 2011 were obtained and compared to the sequences of other available PCV1 isolates. Phylogenetic analyses revealed very low genetic diversity among these viruses, indicating an advanced state in the evolution of PCV1.

## GENOME ANNOUNCEMENT

Porcine circovirus (PCV) was discovered by Tischer et al. (1, 2) as a contaminant of porcine kidney (PK-15) cell lines. PCVs are small nonenveloped viruses with single-stranded circular DNA genomes. The two described genotypes of PCV are present worldwide (3, 4). PCV type 2 (PCV2) is responsible for a wide range of symptoms, called PCV-associated diseases, which have high economic impact and therefore attract much research. In contrast, PCV1 has received less attention due to its being considered nonpathogenic, until recently, when it was shown to cause lesions in experimentally infected pig fetuses (4). Furthermore, PCV1 was detected in several vaccines for human and veterinary use (5–8), probably as a result of the contamination of cell lines and pig-derived enzyme preparations.

Two full-length PCV1 genomes were sequenced, one of them originating from a testicular cell culture from swine in Hungary (PCV-Hun) and the other generated from a field sample taken in the United Kingdom in 1990 and subsequently cultured in swine kidney cells (PCV-Eng_1990). Three clones of each isolate were sequenced. The resulting PCV1 full-length sequences were assembled using the BioEdit sequence alignment editor, and analyses were carried out using the CLC DNA Workbench 6.

Strains PCV-Eng_1990 and PCV-Hun are 1,759 nucleotides long and were compared to the 45 PCV1 complete genome sequences and 6 complete coding sequences (CDS) available in GenBank. Strain PCV-Eng_1990 is identical to a virus genome available under the accession no. JN133302, which was isolated from a stillborn piglet in 1995 in the United Kingdom. Strain PCV-Hun is most similar to the above-mentioned United Kingdom isolate, to strain PCV1-Eng_1990, and to a strain isolated in Australia in 2004 from PK-15 cells (accession no. AY754015), with PCV-Hun differing from these strains by only 4 nucleotides.

Sequence comparisons indicated that the PCV1 isolates present worldwide are highly similar, with the lowest percent identity between any two strains being 97.68%, a result of a 41-nucleotide difference between the genomes of an Australian virus isolated in 2004 and a Chinese strain from 2012 (accession no. AY754012 and KC894933, respectively). Based on a phylogenetic tree constructed using the neighbor-joining method, with a bootstrap value of 1,000, the two newly sequenced strains clustered together with a group of PCV1 sequences consisting of viruses from Asia, North America, Europe, and Australia, isolated between 1995 and 2009 from cell cultures and also from a clinical case of infection (accession no. JN398656, Y09921, AY184287, AY754015, EF493843, CQ449671, and JN133302). This diversity of the geographical origins and sources of isolation, together with the low genetic variation, suggests that the adaptation of PCV1 to the host species is an advanced process, and in contrast to the pathogenic PCV2, the virus is affected by very low selection pressure. This may be because, despite infected animals' showing clinical signs, PCV1 is apathogenic. However, it cannot be excluded that the cause of this genetic uniformity is that most viruses (where the origin of the virus is indicated) are isolated from *in vitro* cell cultures, not from infected animals.

### Nucleotide sequence accession numbers.

Genome sequences of the PCV-Eng_1990 and PCV-Hun strains were deposited in GenBank under accession no. KJ408798 and KJ408799.

## References

[1] Tischer I Rasch R Tochtermann G 1974 Characterization of papovavirus- and picornavirus-like particles in permanent pig kidney cell lines. Zentralbl. Bakteriol. Orig. A 226:153–167 4151202

[2] Tischer I Gelderblom H Vettermann W Koch MA 1982 A very small porcine virus with circular single-stranded DNA. Nature 295:64–66. 10.1038/295064a0 7057875

[3] Rose N Opriessnig T Grasland B Jestin A 2012 Epidemiology and transmission of porcine circovirus type 2 (PCV2). Virus Res. 164:78–89. 10.1016/j.virusres.2011.12.002 22178804

[4] Saha D Lefebvre DJ Ducatelle R Doorsselaere JV Nauwynck HJ 2011 Outcome of experimental porcine circovirus type 1 infections in mid-gestational porcine foetuses. BMC Vet. Res. 7:64. 10.1186/1746-6148-7-64 22018436 PMC3216242

[5] Baylis SA Finsterbusch T Bannert N Blümel J Mankertz A 2011 Analysis of porcine circovirus type 1 detected in Rotarix vaccine. Vaccine 29:690–697. 10.1016/j.vaccine.2010.11.028 21093497

[6] Gilliland SM Forrest L Carre H Jenkins A Berry N Martin J Minor P Schepelmann S 2012 Investigation of porcine circovirus contamination in human vaccines. Biologicals 40:270–277. 10.1016/j.biologicals.2012.02.002 22402185

[7] Quintana J Segalés J Calsamiglia M Domingo M 2006 Detection of porcine circovirus type 1 in commercial pig vaccines using polymerase chain reaction. Vet. J. 171:570–573. 10.1016/j.tvjl.2004.12.008 16624728

[8] Victoria JG Wang C Jones MS Jaing C McLoughlin K Gardner S Delwart EL 2010 Viral nucleic acids in live-attenuated vaccines: detection of minority variants and an adventitious virus. J. Virol. 84:6033–6040. 10.1128/JVI.02690-09 20375174 PMC2876658

